# Study on the Improvement of Quality Characteristics of Pickles During Fermentation and Storage

**DOI:** 10.3390/foods13243989

**Published:** 2024-12-10

**Authors:** Yangyang Chen, Huiyu Gong, Junwei Wang, Tongxun Liu, Mouming Zhao, Qiangzhong Zhao

**Affiliations:** 1 School of Food Science and Engineering, South China University of Technology, Guangzhou 510640, China; giggle_chenyang@163.com (Y.C.); gonghuiyu@163.com (H.G.); jweiwang@163.com (J.W.); txliu@scut.edu.cn (T.L.); femmzhao@scut.edu.cn (M.Z.); 2 Guangdong Food Green Processing and Nutrition Regulation Technology Research Center, Guangzhou 510640, China

**Keywords:** fermentation-promoting peptides, cowpea pickle quality, anti-browning and texture retention

## Abstract

This study investigated the effect of fermentation-promoting peptides (FPPs) on the improvement of the quality of cowpea pickles during fermentation and storage. FPPs were introduced to evaluate their effects on key parameters such as pH, total acidity, nitrite levels, and salinity. FPP accelerated fermentation by stimulating lactic acid bacteria (LAB) activity, leading to a rapid reduction in pH and a stable increase in total acidity. Nitrite accumulation was peaking at 0.56 mg/kg on the 7th day, compared to 1.37 mg/kg in the control, thus enhancing product safety. FPP also improved antioxidant retention, reducing ascorbic acid degradation by 30% and increasing phenolic retention by 15.97% over the control, which is essential for antioxidant capacity and color stability. Texture analysis showed higher hardness preservation in the presence of FPP, in which hardness decreased from 209.70 g to 79.98 g in the FPP group after storage, compared to a decline from 158.56 g to 41.66 g in the control. Additionally, sensory evaluations demonstrated that the FPP group maintained superior flavor, texture, and appearance, with minimized browning due to improved pectin stability. This research presents FPPs as a promising additive for producing high-quality, shelf-stable pickles in line with clean label trends.

## 1. Introduction

Fruits and vegetables play a vital role in human health, and with the world’s growing population and increasing awareness of healthy eating, demand for these essential foods has significantly risen [1]. However, most fresh fruits and vegetables spoil across harvesting, post-harvest management, and retail stages, resulting in substantial losses and waste [2]. Approximately 14% of food globally is lost during the harvest-to-retail process, with fruits and vegetables accounting for as much as 32.99% [3]. Therefore, effective post-harvest strategies are essential to mitigate these issues, often requiring shorter processing times. Pickles represent a traditional preservation technique with over three thousand years of history, involving vegetables such as cowpea, radish, and chili pepper [4,5]. In addition to their sensory qualities, pickles possess various functional properties, including antioxidant, anti-obesity, and anti-cancer effects [6,7,8]. Owing to their unique taste, aroma, and prolonged shelf life, pickles enjoy strong consumer popularity. The global pickle market is projected to reach USD 18.124 billion by 2031, a 34.6% increase from USD 13.461 billion in 2022 [9]. However, during fermentation and storage, pickles may develop undesirable attributes, such as textural softening and browning, which threaten their sensory quality, nutritional value, and economic worth.

Browning poses a significant challenge in the food industry, particularly for fruits and vegetables, as it can negatively affect food quality, especially appearance, thereby reducing consumer acceptance [10]. Browning in fruits and vegetables is generally attributed to both enzymatic and non-enzymatic pathways. Enzymatic browning results from a series of reactions catalyzed by polyphenol oxidase, which oxidizes phenolic compounds into quinones [11]. Non-enzymatic browning encompasses various complex reactions, including the Maillard reaction, ascorbic acid degradation, and phenolic oxidation [12,13,14]. Browning in pickles has been documented in Dongbei Suancai [15,16], Chinese cabbage [17], and radish pickles [18]. Additionally, texture is another essential attribute of pickles, though they may soften and develop an unappealing paste-like texture during fermentation and storage. Previous studies have shown that pectin is closely correlated with the texture of vegetables, and the degradation of pectic substances represents the primary cause of pickle softening [19,20].

Efforts have been made in developing strategies to control browning in food products. Chemical anti-browning agents have been applied to fruits and vegetables, such as sulfites, sulfur-containing organics, ascorbic acid, ethylenediaminetetraacetic acid, and aromatic carboxylic acids [10]. However, these agents pose significant challenges due to health concerns, exemplified by the FDA’s 1986 ban on sulfites and ascorbic acid’s temporary control over browning [21]. Consequently, the development of novel, natural, and safe browning inhibitors is both promising and valuable for fruit and vegetable products. Recent advancements in biotechnology have accelerated the development and utilization of plant protein resources, leading to the identification and characterization of numerous bioactive peptides [22,23]. Soybean peptides constitute low-molecular-weight peptides obtained through enzymatic hydrolysis of soybean protein, followed by specialized processing [24]. Since the mid-19th century, researchers have suggested that soy peptides promote microbial growth and reproduction [25]. For microorganisms, soy peptides are a highly valuable nitrogen source, even superior to free amino acids, enhancing their growth, development, and metabolism [26]. Studies on fermentation promotion primarily focus on short-term fermented products like yogurt and beer [27]. Research has also shown that soy protein hydrolysates can increase the acid production capacity of lactic acid bacteria, reduce fermentation time, and improve the storage quality of dairy products [28].

Cowpea (*Vigna unguiculata*) is a nutrient-dense crop widely cultivated for its heat and drought tolerance, providing a high-quality dietary protein source in regions with adverse environmental conditions and positively contributing to alleviating global malnutrition [29,30]. Cowpea benefits human health in various ways, offering anti-cancer, anti-hypertensive, cardiovascular disease-preventing, anti-diabetic, and anti-inflammatory properties due to its rich bioactive compound content [31]. Based on these advantages, cowpea pickles have become a popular fermented vegetable in China, with significant potential for further development [32]. Nitrates in pickled vegetables can also pose potential food safety issues, and the imagination of nitrite accumulation in cowpea during fermentation has been widely reported [33,34]. The mixed inoculation of *Weissella cibaria* and *Lactobacillus plantarum* was used to improve the quality of pickled cowpeas, reducing the concentrations of nitrates and biogenic amines by 5–12% and 6–12%, respectively [35]. Studies have also shown that inoculation of lactic acid bacteria or accelerated acid production at the initial stage of fermentation can significantly inhibit the formation of nitrite [36]. Furthermore, the oxidation of phenolic compounds has also been shown to be one of the causes of browning in pickles, though this has been overlooked in pickled cowpeas [37]. Therefore, this study focuses on cowpea pickles and investigates the effects of soybean-derived fermentation-promoting peptides (FPPs) on the improvement of the quality of cowpea pickles during fermentation and storage, particularly in terms of browning and texture. The aim is to propose novel quality improvement strategies for fruit and vegetable post-processing and pickle production, contributing to efforts addressing environmental issues caused by loss and waste.

## 2. Materials and Methods

### 2.1. Materials

The cowpeas were sourced from Hongbin Food Co., Ltd. (Honghe Hani and Yi Autonomous Prefecture, China). 5-HMF was obtained from Sigma Aldrich Co. (St. Louis, MI, USA). Potassium dihydrogen phosphate, metaphosphoric acid, ascorbic acid, methanol, glacial acetic acid, and acetonitrile were supplied by Sinopharm Chemical Reagent Co., Ltd. (Shanghai, China). The soybean-derived FPP was developed by the School of Food Science and Engineering, South China University of Technology (Guangzhou, China).

### 2.2. Preparation of Cowpea Pickles

Cowpeas were washed, and approximately 8 kg of high-quality cowpeas were placed in treated 20 L fermentation jars with 10 L of a 6% salt solution. Each jar was inoculated with 200 mL of a Lactobacillus plantarum suspension, water-sealed, and fermented at 28 °C. Lactobacillus plantarum strain HP1438, isolated by Hongbin Food Co., Ltd., was used as the dominant representative strain in pickle fermentation. Lactobacillus plantarum preserved in glycerol was prepared for fermentation through activation, amplification, and dilution procedures.

### 2.3. Determination of Microorganisms

The method by Du, Zhang [38] was used to determine the total bacterial count and lactic acid bacteria in cowpea pickles. Microorganism numbers were determined via plate counting, using agar medium and MRS agar medium. Culture conditions were set at 37 °C for 48 h for total bacteria and 37 °C for 72 h for lactic acid bacteria (LAB). Data are expressed in colony-forming units (cfu), with results presented as cfu/g.

### 2.4. Determination of pH, Total Acidity, Nitrite, and Salinity

The pickles and fermentation liquid were homogenized at a 1:1 (*w/w*) ratio to obtain a pickle homogenate, which was then centrifuged at 5000× *g* for 5 min, with pH measured using a micro pH electrode [39]. Total acidity was determined by boiling 5.0 g of pickle homogenate with 50 mL of distilled water, soaking for 30 min, filtering, and titrating with NaOH (0.05 mol/L) to a pH of 8.2 ± 0.2 [40]. Salinity was assessed through AgNO_3_ titration (0.10 mol/L) until a brick-red precipitate formed, using potassium chromate (0.5 mol/L) as an indicator, and calculated from the titrated chloride ions [41]. Nitrite content was determined spectrophotometrically: 10.0 g of cowpea pickle homogenate was mixed with 100 mL distilled water and 5 mL ammonia buffer solution (pH 9.6–9.7), shaken for 30 min, and treated with 2 mL potassium ferrocyanide solution (150 g/L) and 2 mL zinc sulfate solution (300 g/L), then filtered after standing for 5 min. Absorbance was measured at 219 nm, and a standard curve was established [42].

### 2.5. Amino Acid Nitrogen and Total Sugar

A 10.0 g sample of pickle homogenate was mixed with 70 mL distilled water and titrated with NaOH solution (0.05 mol/L) to a pH of 8.2. After adding 10 mL formaldehyde solution (38%, *v*/*v*), the mixture was further titrated with NaOH solution to a pH of 9.2 to record volume V1. Using 80 mL distilled water as a blank, NaOH was titrated to a pH of 9.2 to record volume V2. The difference between V1 and V2 represented the content of amino acid nitrogen [43]. For total sugar determination, 5.0 g of pickle homogenate was boiled with 25 mL distilled water for 30 min, cooled, and filtered. The supernatant was analyzed by the 3,5-dinitrosalicylic acid method [44].

### 2.6. Total Phenol and Total Flavonoid

The Folin–Ciocalteu method was employed to determine the total phenol content in cowpea pickles. Pickle homogenate and 80% methanol solution were mixed at a 1:5 (*m*/*v*) ratio, ultrasonically extracted for 30 min, and centrifuged at 10,000× *g* for 10 min to obtain the supernatant [45]. In total, 1 mL of supernatant was added to 1 mL Folin–Ciocalteu reagent, mixed well, and reacted in the dark for 5 min. Then, 3 mL sodium carbonate solution (7.5%) was added, mixed, and reacted in the dark for 1 h. Absorbance was measured at 765 nm, and a standard curve was prepared using gallic acid. Total flavonoid content was determined by mixing 1 mL supernatant with methanol (80%) and sodium nitrite (5%), reacting for 6 min, then adding aluminum chloride (10%) and NaOH (4%) and reacting for 30 min. Absorbance was measured at 510 nm, with a standard curve prepared using rutin [46].

### 2.7. 5-HMF Content

The 5-HMF content was determined via high-performance liquid chromatography (HPLC-2695, Waters, Milford, MA, USA). The pickle homogenate was diluted tenfold with methanol solution (10%), filtered through a 0.22 µm filter, and analyzed [15]. Chromatographic conditions included a TC-C18 column (4.6 mm × 250 mm, 5 µm) at 30 °C, with a mobile phase of acetic acid solution (1%) and methanol (90:10), a flow rate of 0.6 mL/min, and detection at 280 nm.

### 2.8. Ascorbic Acid

Ascorbic acid extraction and determination followed a slightly modified method by Yang, Fan [47]. The pickle homogenate was diluted tenfold with distilled water, extracted at 75 °C for 20 min, cooled, and centrifuged at 5000× *g* for 20 min. The supernatant was filtered through a 0.22 µm filter and analyzed. HPLC conditions included an AQ-C18 column (4.6 mm × 100 mm, 2.7 µm) at 27 °C, with a mobile phase of KH2PO4 solution (0.01 mol/L) and methanol (97:3), a flow rate of 0.6 mL/min, and detection at 210 nm.

### 2.9. Protopectin and Water Soluble Pectin

Protopectin and pectin extraction and determination procedures followed a slightly modified method by Zheng, Zhang [48]. In total, 5.0 g of cowpea pickles were treated with 95% ethanol, heated in a boiling water bath for 30 min, and filtered. The precipitate was heated with 50 mL ultrapure water at 50 °C for 2 h, then filtered, with the precipitate representing protopectin and the filtrate representing water-soluble pectin. Pectin content was determined by the carbazole colorimetric method, with a standard curve prepared using galacturonic acid.

### 2.10. Color Measurement

The color measurement was slightly modified according to the method of Jha, Chevallier [49]. The color difference (L*, a*, and b* values) of cowpea pickles was measured using a colorimeter (CR-400, Konica Minolta, Chiyoda City, Japan), calibrated with a white standard plate (L* = 96.15, a* = 0.12, b* = 1.73).

### 2.11. Browning Index

Based on Wang, Zhang [16], pickle homogenate was mixed with ethanol (67%) at a 1:9 (*m*/*v*) ratio, extracted at 35 °C for 2 h, and centrifuged at 10,000× *g* for 15 min. The absorbance of the supernatant was measured at 420 nm.

### 2.12. Texture Analysis

The texture of cowpea pickles was slightly modified and analyzed using a texture analyzer and P2/N probe (TA-XT plus, Stable Micro Systems, Godalming, UK) according to the method provided by Zhao, Ge [50]. The pre-test, test and post-test speeds were 1.0, 1.0 and 5.0 mm/s, respectively, and the puncture distance was 10 mm.

### 2.13. Sensory Analysis

In total, 20 professional food technicians evaluated the sensory qualities of fermented and stored cowpea pickles. Scoring criteria and descriptions are shown in Table S1.

### 2.14. Data Analysis

Data analysis was conducted using SPSS software (SPSS26, IBM Corporation, Chicago, IL, USA), with results expressed as mean ± standard deviation using one-way analysis of variance (ANOVA). All figures were created using Origin software (2024, OriginLab, Northampton, MA, USA).

## 3. Results and Discussion

### 3.1. Effect of FPP on Key Chemical Indexes of Cowpea Pickle During Fermentation

#### 3.1.1. Changes in Microorganisms

**Figure 1:**
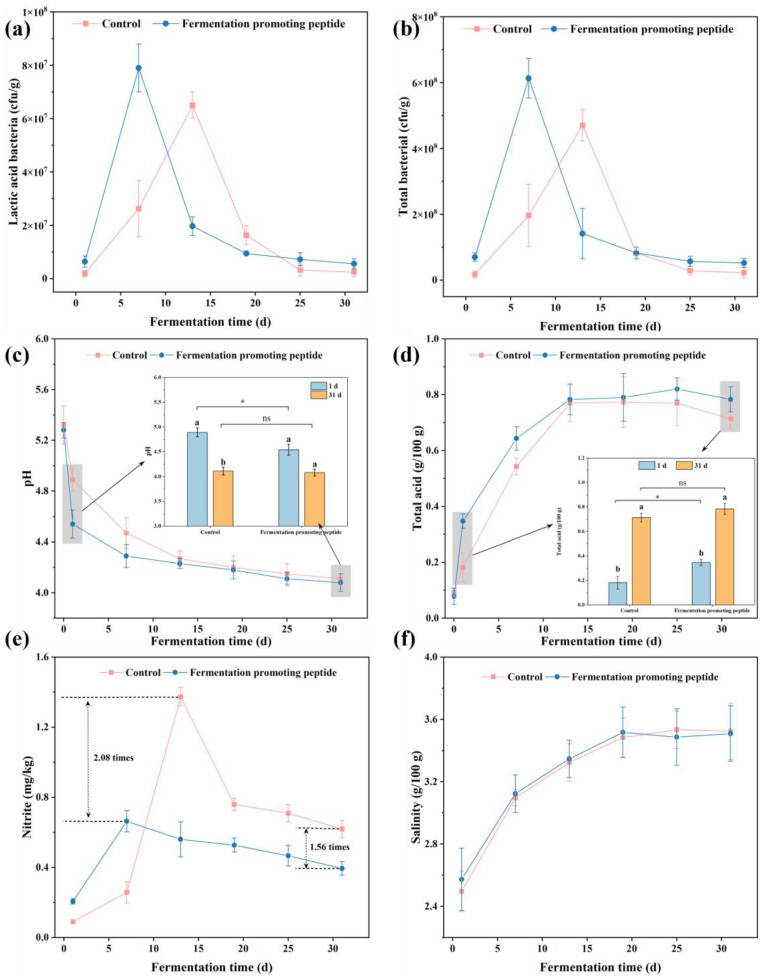
Changes in lactic acid bacteria (**a**), total bacterial (**b**), pH (**c**), total acid content (**d**), nitrite (**e**), and salinity (**f**) of cowpea pickles during fermentation. a, b: significant differences between different groups under the same treatment; *: significant differences under different treatments within the same group (*p* < 0.05); ns: not significant.

Figure 1a,b present the LAB and total bacterial counts in the control and FPP groups during the fermentation of cowpea pickle. As shown in Figure 1a, LAB counts increased markedly in both groups, peaking on the 13th day in the control and on the 7th day in the FPP group. Compared to the control (6.5 × 10^7^ cfu/g), the peaking count of LAB was significantly higher in the FPP group (7.9 × 10^7^ cfu/g). After reaching their peaks, LAB counts in both groups decreased and stabilized, with the FPP group maintaining a higher count throughout the fermentation period. Figure 1b illustrates total bacterial counts, which followed a similar trend as that of LAB. The total bacterial count of the FPP group peaked at approximately 6.1 × 10^8^ cfu/g on the 7th day, while that of the control peaked at 4.7 × 10^8^ cfu/g on the 13th day. Total bacterial counts then gradually decreased in both groups, with the FPP group again maintaining greater microbial stability throughout fermentation.

LAB plays an essential role in vegetable fermentation by producing lactic acid, reducing pH, and inhibiting spoilage organisms, contributing to the safety and flavor development of fermented products [5]. Enhanced LAB growth in the FPP group suggests that FPP supports microbial activity, potentially by providing additional nutrients or functioning as a growth stimulant. This observation aligns with previous studies, showing that protein hydrolysates and bioactive peptides can enhance LAB vitality and activity, enabling a faster and more efficient fermentation process [51,52]. Furthermore, LAB reached the peaking counts earlier in the FPP group compared to the control, likely due to the effect of FPP on microorganism growth and activity during fermentation. FPP may supply additional nutrients or growth factors to stimulate early LAB proliferation. Peptides are known to support bacterial metabolism by serving as nitrogen sources that are readily absorbed, promoting faster cell division and metabolic activity [53]. This is also reflected in the faster pH decrease in the FPP group due to enhanced LAB activity. The rapid acidification created an optimal environment for LAB growth in the early fermentation process, which can also inhibit spoilage organisms. Faster pH reduction allowed LAB to preferentially outcompete other bacteria, leading to higher and earlier peaks in LAB populations. Bioactive peptides, such as FPP, may stimulate specific metabolic pathways in LAB, improving their fermentation capabilities, including carbohydrate fermentation efficiency to produce lactic acid, thereby supporting faster growth and increased LAB counts early in the process [51]. As shown in Figure 1b, the total bacterial count was higher in the FPP group, further affirming the beneficial effect of FPP on microbial stability. Bacterial counts in both groups declined sharply after peaking (7th and 13th day) as nutrient depletion and organic acid accumulation created a less favorable environment for bacterial survival. However, the FPP group experienced a slower decline, indicating that FPP contributes to maintaining a stronger microbial population in the later fermentation stages. Overall, these results suggest that FPP enhances the fermentation process by promoting higher LAB activity and maintaining greater microbial stability, positively affecting both the fermentation procedure and the quality of cowpea pickles.

#### 3.1.2. pH and Total Acid

pH and total acid are essential quality parameters for pickles, significantly impacting sensory quality, influencing microbial growth, and serving as indicators of pickle ripeness and spoilage [54]. Figure 1c,d evaluate the influence of FPP on pH and total acid content in cowpea pickles during fermentation, revealing distinct differences in fermentation kinetics between the control and FPP groups. As shown in Figure 1c, the pH of the control gradually decreased from 5.3 to 4.1 during the fermentation period, however, that of the pH in the FPP group declined more rapidly, dropping from 5.3 to around 4.2 within the first 7 days and maintaining this level until the end of fermentation. These trends were further highlighted by the illustrations in Figure 1c, showing that both groups had significantly higher pH values on the 1st day compared to the 31st day. Additionally, on the 1st day, the pH of the control group was 4.9, significantly higher than that of the FPP group. However, by the 31st day, there was no significant difference. Figure 1d illustrates the total acid content of cowpea pickles over the same fermentation period. In the control group, total acid content steadily increased from 0.09 g/100 g on the 1st day to 0.18 g/100 g, peaking at 0.77 g/100 g on the 13th and remaining stable thereafter. The FPP group showed a more rapid increase in total acid, rising sharply from 0.08 g/100 g on the 1st day to 0.35 g/100 g and peaking at 0.78 g/100 g on the 13th day, maintaining stability for the fermentation period. Figure 1d provides a more detailed comparison of the total acid content between the control and FPP groups on the 1st and 31st day. The total acid content of both groups was significantly higher on the 31st day than on the 1st day. No significant difference in the total acid content was observed between the control and FPP groups. Nevertheless, on the 1st day, the FPP group had a significantly higher total acid level than the control (*p* < 0.05).

The experimental data indicate that FPP significantly enhanced the early-stage fermentation of cowpea pickles. The pH decreased more rapidly in the FPP group, suggesting enhanced microbial activity, especially in the growth and metabolism of lactic acid bacteria (LAB). LAB is well known for its crucial role in vegetable fermentation, primarily through organic acid production, such as lactic acid. The lower and rapidly declining pH observed on the 1st day in the FPP group suggests that FPP created a more favorable environment for LAB, possibly by supplying essential amino acids or acting as peptides that enhance metabolism. This aligns with previous research showing that peptides can stimulate bacterial growth and acid production, thereby accelerating the fermentation rate [55,56]. This conclusion is further corroborated by total acid, which was significantly higher in the FPP group, with a sharp increase observed during the first 7 days of fermentation. This higher acid content contributes to a more stable environment, as increased lactic acid levels inhibit the growth of spoilage microorganisms, thus improving the safety and shelf life of fermented products [57]. The stable content of total acid in the later stages of fermentation also suggests that bacterial metabolic activity entered a stationary phase, possibly due to the depletion of fermentable substrates. The optimal maturation stage for pickles is defined as a total acid level of 0.5–0.9 g/100 g or a pH of 4.2–4.5, and the FPP group reached this standard on the 7th day of fermentation, which was faster than that of the control group on the 13th day, indicating that FPP can effectively expedite the fermentation process [58].

#### 3.1.3. Nitrite and Salinity Changes

Nitrite is a widely known harmful compound in pickles capable of forming carcinogenic nitrosamines, which can pose cancer risks in the digestive system [59]. Figure 1e,f show the changes in nitrite content and salinity in the control and FPP groups throughout the fermentation period. Nitrite levels in the control group increased sharply in the initial stages of fermentation, peaking at 1.37 mg/kg on the 13th day. However, nitrite accumulation was significantly lower in the FPP group, peaking at only 0.56 mg/kg on the 7th day (Figure 1e). After reaching the peaking content, nitrite levels decreased in both groups, stabilizing at 0.62 mg/kg in the control group and 0.39 mg/kg in the FPP group on the 31st day, which was 1.56 times lower in the FPP group than in the control. Figure 1f indicates that salinity levels in both the control and FPP groups gradually increased from 2.49 g/100 g on the 1st day to 3.32 g/100 g on the 13th day, continuing to increase slightly before stabilizing around 3.5 g/100 g by the 31st day, no significant difference was observed between the two groups.

Nitrite changes indicate that the addition of FPP significantly inhibited nitrite accumulation during the initial stages of fermentation. Nitrite formation in fermented vegetables typically results from the conversion of nitrate to nitrite by Gram-negative bacteria through assimilation or denitrification processes [42]. The lower nitrite accumulation in the FPP group suggests that FPP either directly inhibited Gram-negative bacteria or promoted LAB growth, accelerating acidification and thereby resulting in reduced nitrite levels. FPP can support the rapid growth and dominance of LAB, and LAB metabolism produces substantial lactic acid, which lowers pH and inhibits the growth of Gram-negative bacteria, thereby reducing nitrite production [60]. Previous studies have similarly demonstrated that rapid acidification during fermentation can inhibit nitrate-reducing bacterial activity, thus reducing nitrite formation [61]. The salinity results reveal a similar trend of increasing and stabilizing salt concentration in both groups during fermentation (Figure 1f). Salinity plays a crucial role in vegetable fermentation, affecting microbial activity and the final product texture. The comparable salinity patterns in both groups suggest that FPP does not significantly alter salt uptake or distribution during fermentation, which is essential for maintaining product safety and quality during storage [57].

### 3.2. Effect of FPP on the Color Quality of Cowpea Pickle During Fermentation

#### 3.2.1. Ascorbic Acid, Total Phenols and Total Flavonoids

Ascorbic acid degradation is a common issue in fermented vegetables, often exacerbated by prolonged storage, degradation in both aerobic and anaerobic environments, and the formation of intermediate products that combine with amino acids to produce brown pigments, ultimately causing browning [62]. Flavonoids and phenolic compounds are key antioxidants that affect the color, flavor, and nutritional value of fermented vegetables, as well as important contributors to non-enzymatic browning [37]. The effects of FPP on ascorbic acid, total flavonoid, and total phenol contents during fermentation and accelerated storage of cowpea pickles are shown in Table 1. The ascorbic acid content in the control and FPP groups decreased by 30% and 20% during fermentation, respectively, and it fell below the detection limit after storage. Additionally, the ascorbic acid content in the FPP group was significantly higher than that of the control on the 1st and 31st day of fermentation, with less loss of 16.67% and 30.00%, respectively. Table 1 shows that total flavonoid content significantly decreased in both groups, with the control group declining by 20.52% during fermentation and further declining to 41.49 µg/g after storage. A higher flavonoid level was consistently maintained for the FPP group, with only an 11.04% decrease after fermentation and a final value of 43.09 µg/g after storage. The total phenol content in both groups is presented in Table 1. Compared to the control, significantly higher total phenol content was observed for the FPP group after fermentation. Both groups experienced a significant decrease after storage, with the control reaching 83.02 µg/g, which was significantly lower than that of the FPP group (97.52 µg/g).

FPP significantly enhanced the retention of ascorbic acid, flavonoids, and phenols during fermentation and storage. The slower degradation rate of ascorbic acid in the FPP group suggests that FPP may enhance the stability of ascorbic acid or reduce oxidative stress, thereby maintaining its content. This is consistent with findings that bioactive peptides possess antioxidant properties, scavenging free radicals and protecting ascorbic acid from degradation [55,63]. The decrease in total phenol and flavonoid content during fermentation and storage may be due to the acidic environment of fermentation causing phenolic structure rearrangement, self-polymerization, or interactions with other macromolecules, which reduce extractability [64]. The higher retention of these compounds in the FPP group demonstrates the ability of peptides to inhibit oxidative degradation during fermentation and storage [65]. Similar effects have been observed in corn protein hydrolysates, which can effectively inhibit lipid peroxidation in pork powder, enhancing product stability during transportation and storage, thereby extending food shelf life [66]. Broad bean protein hydrolysates, containing a variety of antioxidant peptides, improve apple juice quality by scavenging free radicals or chelating metal ions [67]. In conclusion, FPP not only enhances the fermentation process but also contributes to the preservation of essential antioxidant compounds during fermentation and storage, making FPP a promising green strategy for improving the quality and shelf life of fermented vegetables.

#### 3.2.2. Amino Acid Nitrogen, Total Sugar and 5-HMF Content

Table 2 shows the effects of FPP on amino acid nitrogen, total sugar, and 5-HMF levels in cowpea pickles during fermentation and storage. As shown in Table 2, the content of amino acid nitrogen levels in all groups increased significantly during fermentation but decreased sharply during storage. Amino acid nitrogen content in the FPP group was 15.48% higher than the control on the 1st day of fermentation; however, it was 23.23% lower than that of the control on the 31st day. No significant difference in the content of amino acid nitrogen between groups was observed after storage. As shown in Table 2, total sugar content decreased over time, with the FPP group showing significantly lower levels on the 1st and 31st day of fermentation, which was approximately 11.01% and 30.83% lower than that of the control, respectively. After accelerated storage, no significant difference in total sugar levels was observed between the FPP and control groups. Table 2 also illustrates that 5-HMF was undetectable in either group on day 1 of fermentation, and it was approximately 0.1 µg/g on the 31st day. After storage, 5-HMF content increased significantly in both groups, with the FPP group 24.87% lower than the control.

Amino acid nitrogen serves as an important indicator of protein hydrolysis during fermentation and contributes to flavor formation in fermented foods. The increase in amino acid nitrogen content during fermentation is likely due to protein breakdown into free amino acids, which increases amino nitrogen levels early in the fermentation process [68]. The content of amino acid nitrogen in the FPP group was significantly higher than the control on the 1st day of fermentation, likely due to FPP-enhanced microbial activity which facilitated protein breakdown into amino acids. However, the content of amino acid nitrogen decreased after peaking, which was due to the fact that microbes utilize amino acids as substrates. The enhanced microbial activity of FPP likely accelerated this process, resulting in a significantly lower content of amino acid nitrogen in the FPP group on the 31st day compared to the control. Total sugar, an essential substrate for microbial growth during fermentation, was significantly lower in the FPP group than in the control on the 1st day, indicating more vigorous fermentation activity at this stage. The FPP group also had significantly lower total sugar content on the 31st day, reflecting enhanced microbial activity and a more intensive fermentation process, which led to greater sugar consumption.

The Maillard reaction is a recognized pathway of non-enzymatic browning, where amino acids and reducing sugars undergo rearrangement, dehydration condensation, and polymerization to form melanin-like substances, resulting in browning and quality changes [37]. 5-HMF, an important Maillard reaction intermediate, reacts with amines in the final stages to form melanoidins and is often used as a marker for non-enzymatic browning [69,70]. During fermentation, the content of 5-HMF levels in both the FPP and control groups remained extremely low, indicating minimal Maillard reaction activity at this stage. Reports suggest that the Maillard reaction primarily contributes to browning during storage [12,71]. After storage, the content of 5-HMF content in all groups reached approximately 20 µg/g, reflecting a pronounced Maillard reaction during this period. The content of 5-HMF level in the FPP group was significantly lower than the control, likely due to lower amino acid nitrogen and total sugar concentrations and possibly the antioxidant properties of FPP. Although FPP did not directly inhibit the Maillard reaction during fermentation, it indirectly inhibited the formation of 5-HMF formation by reducing available reaction substrates, such as sugars and amino acids, thereby preserving cowpea pickle quality. This protective effect is essential for maintaining product quality during storage, as 5-HMF accumulation is commonly associated with flavor deterioration and reduced shelf life. In summary, FPP effectively reduces 5-HMF formation by regulating amino acid nitrogen and sugar consumption, thereby enhancing the overall quality and shelf life of cowpea pickles.

#### 3.2.3. Browning Index and Color Difference

**Figure 2:**
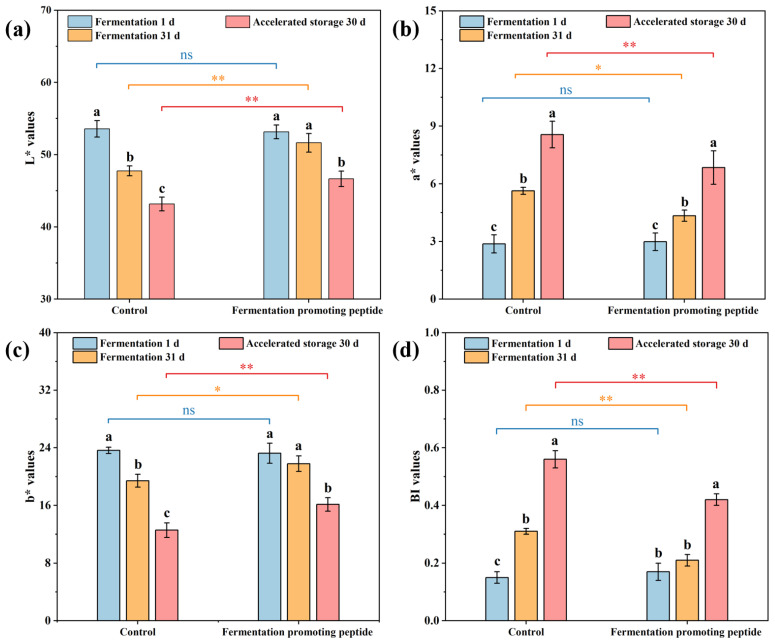
Effects of different treatments on L* value (**a**), a* value (**b**), b* value (**c**) and BI value (**d**) of cowpea pickles during fermentation and storage. *: significant differences under different treatments within the same group (*p* < 0.05); **: significant differences under different treatments within the same group (*p* < 0.01); ns: not significant; a–c: significant differences between different groups under the same treatment.

Color is a critical sensory indicator of food quality, and the CIELAB color system was used to characterize color changes in cowpea pickles during fermentation and storage, as represented by L*, a*, and b* values in Figure 2a–c. The L* value in both control and FPP groups decreased significantly from the 1st day to the 31st day of fermentation, with a more pronounced decrease in the control group (Figure 2a). After storage, the L* value further decreased, with the control group reaching the lowest L* value (43.17) and the FPP group maintaining a relatively higher L* value (46.65). In Figure 2b, the a* value of both groups increased significantly during fermentation and storage, with the FPP group displaying a significantly lower a* value than the control at the end of both phases. Figure 2c shows that the b* value followed a trend similar to the L* value, decreasing over time and remaining significantly higher in the FPP group than in the control. Browning index (BI), defined as the absorbance at 420 nm, is commonly used to characterize and evaluate browning development in vegetables and pickles [15]. Figure 2d illustrates that BI value in both groups increased significantly after storage, with the control group showing a significantly higher BI value (0.56) compared to the FPP group (0.42). Additionally, the control group displayed a notable increase in BI during fermentation, surpassing the FPP group by day 31, whereas no significant difference was observed on the 1st day.

In the CIELAB system, L* represents lightness, a* indicates the red-green color axis, and b* signifies the yellow-blue axis [72]. The slower decrease in L* value in the FPP group suggests that FPP effectively inhibited browning and preserved product brightness, a key attribute for consumer appeal. The increase in a* value and the decrease in b* value in the control group, reflecting intensified red and reduced yellow hues, imply more pronounced non-enzymatic browning, likely resulting from higher levels of Maillard reaction products [73]. Furthermore, the acidic pH of the fermentation and storage environment can promote the chemical oxidation of phytochemicals, generating brown o-quinones that diminish green hues [74]. Flavonoids are also implicated in color changes in yellow foods, with studies linking them to the golden-yellow hue of traditional, non-salted Suancai [75]. The smaller changes in a* and b* values in the FPP group suggest that FPP moderates these effects, potentially by enhancing LAB activity and metabolism. LAB not only produces organic acids and reduces pH to inhibit browning but also generates metabolites with strong metal-ion-chelating and antioxidant properties that effectively limit browning [76]. The BI value serves as a comprehensive indicator of overall browning, with higher values reflecting more extensive non-enzymatic browning. The sharp increase in BI value in the control group after storage corresponds to the higher a* and lower L* values, indicating significant browning and color degradation. Nevertheless, the FPP group maintained a significantly lower BI value, suggesting that FPP may inhibit browning. This effect can be attributed to LAB metabolites and bioactive peptides, which scavenge free radicals and prevent the formation of melanin-like pigments responsible for browning. Various LAB metabolites, such as acids, ketones, carbon dioxide, hydrogen peroxide, and diacetyl, demonstrate strong metal-ion-chelating and antioxidant properties, effectively inhibiting browning [77]. LAB has also shown the ability to protect the color of pear juice by inhibiting polyphenol oxidase activity, enhancing phenol and ascorbic acid retention, and reducing enzymatic and non-enzymatic browning [78]. Similarly, LAB treatment has been shown to improve post-harvest quality in lotus root by significantly reducing color loss [79]. FPP group can maintain a high L* value and low BI value, which supports the conclusion that FPP protects the color quality of cowpea pickles during extended storage.

### 3.3. Changes in Texture Characteristics and Pectin Content

**Figure 3:**
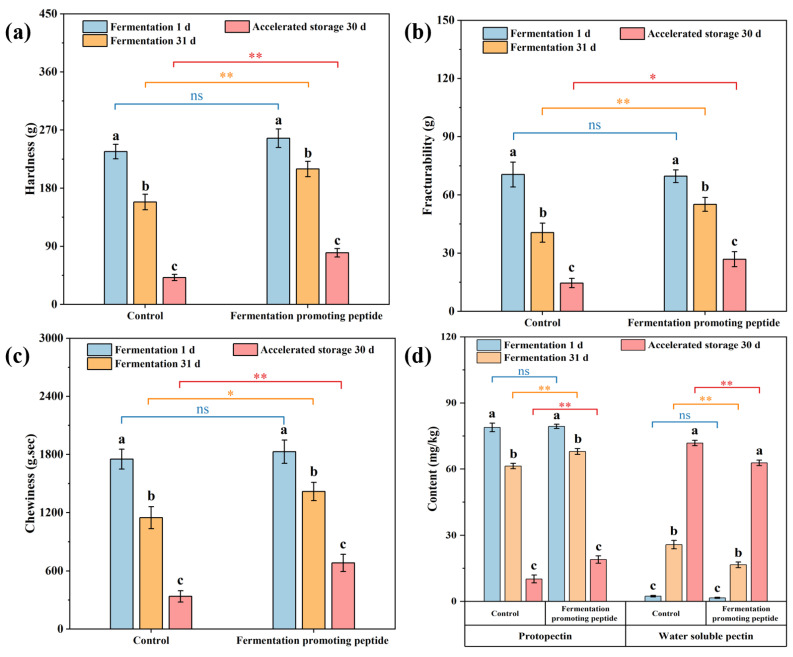
Texture changes, hardness (**a**), fracturability (**b**), chewiness (**c**) and pectin content (**d**) of cowpea pickles during fermentation and storage with different treatments. *: significant differences under different treatments within the same group (*p* < 0.05); **: significant differences under different treatments within the same group (*p* < 0.01); ns: not significant; a–c: significant differences between different groups under the same treatment.

Texture plays a crucial role in food selection, intake, and satiety. Consumers’ sensory perceptions are influenced by the textural, visual, and tactile properties of packaging and food products, which shape expectations and influence purchase intent [80]. Figure 3a–d illustrates changes in textural properties and pectin content in cowpea pickles during fermentation and storage. As shown in Figure 3a, hardness decreased from 236.63 g to 158.56 g in the control group during fermentation and dropped further to 41.66 g after storage. However, the FPP group experienced a much slower decrease in hardness, which maintained a higher value than the control group both during fermentation (209.70 g) and after storage (79.98 g). Figure 3b shows the fracturability, where a similar trend as hardness was observed; the control group showed greater friability loss (final reduction to 14.60 g) compared to the FPP group (final reduction to 26.88 g), indicating better textural integrity in the FPP group. Figure 3c shows chewiness; it followed a similar pattern, with less loss in the FPP group than in the control (final 682.74 g·s and 337.58 g·s, respectively). Figure 3d displays the content of protopectin and water-soluble pectin; the protopectin decreased and the water-soluble pectin increased in both groups during fermentation and storage. On the 31st day of fermentation and after storage, the content of protopectin content in the FPP group was significantly higher than the control; however, the content of water-soluble pectin in the FPP group was significantly lower.

Hardness, fracturability, and chewiness are the key textural properties, they tended to decrease during vegetable fermentation, mainly due to protopectin decomposition. Studies have shown that pectin breakdown in vegetable cell walls is a primary cause of pickle softening [19,81]. Protopectin primarily exists in the primary cell wall and has good adhesion, combining with fibers to form pectin fibers that provide strength and density to plant tissues. Protopectin can be converted to soluble pectin through the action of protopectinase or under acidic conditions [82]. The addition of FPP to cowpea pickles significantly helped preserve pickle texture during fermentation and storage. This may be attributed to the enhancing microbial activity induced by FPP, particularly LAB, which produces organic acids, stabilize cell walls, and inhibit excessive pectin degradation. Li, Bai [79] reported that LAB improved postharvest texture in lotus root, notably enhancing hardness, chewiness, elasticity, and adhesiveness. Zhang, Yu [83] found that LAB fermentation inhibited the activity of microorganisms and enzymes such as polyphenol oxidase, peroxidase, and phenylalanine ammonia lyase in freshly cut lotus root, slowing physiological reactions and maintaining tissue hardness. Encapsulated Lactobacillus plantarum has also been applied to freshly cut apples to slow declines in physical and chemical quality, increasing the antioxidant enzyme activity that scavenges reactive oxygen species [84]. As shown in Figure 3d, the level of protopectin decreased in both groups during fermentation, but the FPP group retained more protopectin than the control group. Conversely, water-soluble pectin associated with softening increased more in the control group, indicating loss of texture, while the increase in water-soluble pectin in the FPP group was more controlled. This may be due to the LAB dominance in the presence of FPP, which could inhibit other microorganisms that decompose protopectin, and the low pH from acid production inhibited protopectinolytic enzyme activity [79]. Additionally, bioactive peptides may enhance plant cell walls by interacting with pectin-degrading enzymes, thus reducing softening in fermented products [85]. The higher content of water-soluble pectin in the control group reflects greater cell wall degradation, resulting in more pronounced texture loss. In general, the present results indicated that FPP effectively reduced texture degradation in cowpea pickles by slowing pectin hydrolysis and retaining essential structural components like protopectin.

### 3.4. Sensory Evaluation

**Figure 4:**
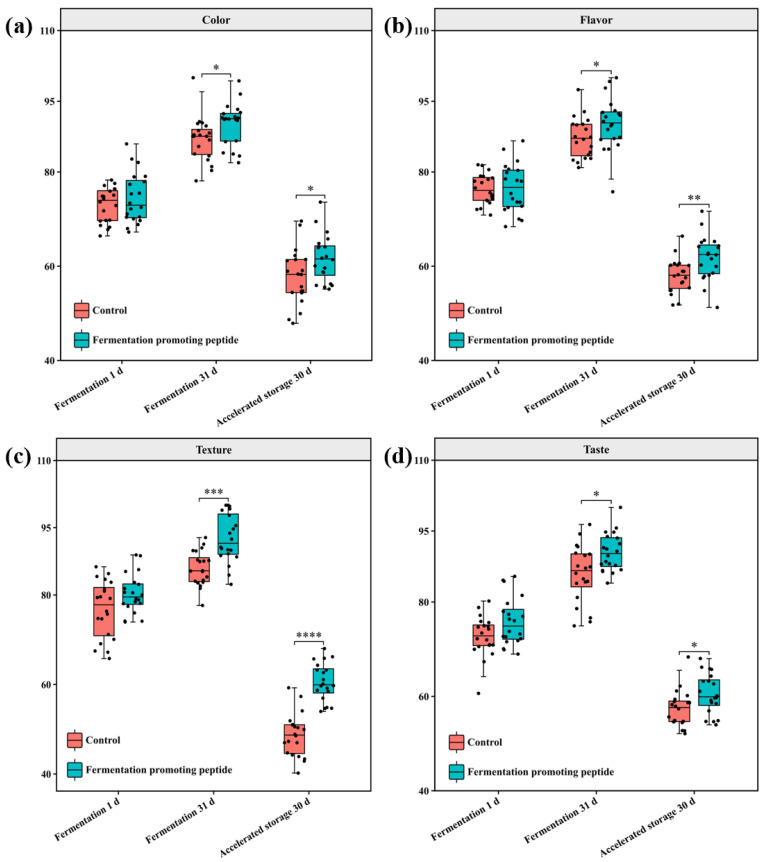
Sensory evaluation results, color (**a**), flavor (**b**), texture (**c**) and taste (**d**) of cowpea pickles with different treatments during fermentation and storage. *: *p* < 0.05; **: *p* < 0.01; ***: *p* < 0.001; ****: *p* < 0.0001.

Figure 4a–d illustrates sensory evaluation results for cowpea pickles in the FPP and control groups, assessing key sensory characteristics including color (a), flavor (b), texture (c) and taste (d). Sensory scores improved across all groups during fermentation, with the FPP group consistently outperforming the control group across all four sensory parameters. After storage, sensory scores decreased in both groups, and the FPP group maintained higher scores than the control, especially in texture (Figure 4c) and flavor (Figure 4b). Regarding color, the FPP group retained better visual appeal than the control (Figure 4a). Similarly, the FPP group exhibited a higher texture score (Figure 4c), indicating higher hardness, while the control group showed marked deterioration after storage. Taste followed a similar trend, with the FPP group sustaining higher sensory scores than the control group after both fermentation and storage (Figure 4d).

Results indicated that FPP contributed to the improvement of sensory quality improvements in cowpea pickles during fermentation and storage. For color, pickles treated with FPP maintained a more appealing visual appearance, likely attributed to the ability of peptides to inhibit oxidative browning reactions, reducing discoloration in fermented vegetables [86,87]. Additionally, LAB dominance also supported by FPP contributes to browning inhibition [76]. The improved flavor in the FPP group may be attributed to the preservation of volatile aroma compounds and a reduction in unwanted flavors. Studies suggest that bioactive peptides regulate the microbial environment, thereby reducing spoilage and enhancing the production of flavor-enhancing metabolites, such as amino acids and organic acids [55]. This texture enhancement may be due to the direct or indirect role of FPP in pectin stabilization, which reduces the degree of pectin decomposition into water-soluble pectin, thereby inhibiting the softening of fermented vegetables. As shown in a previous analysis, the FPP group demonstrated superior chewiness and fracturability, aligning with these sensory evaluations. The FPP group also showed considerable improvement in taste, with enhanced freshness and sweetness attributed to the retention of amino acids and sugars which essentially contributed to the flavor profile of fermented products. On the whole, FPP enhanced the sensory quality of cowpea pickles, particularly in terms of texture, flavor, and color. These benefits may be attributed to FPP effects on microbial activity, pectin preservation, and the regulation of browning and oxidation reactions during fermentation and storage.

## 4. Conclusions

This study demonstrated the significant impact of FPP on enhancing the quality, safety, and sensory appeal of cowpea pickles during both fermentation and storage. FPP produced optimal results across multiple quality parameters, which establishes FPP as an effective functional additive in pickle production. FPP accelerated fermentation by boosting LAB, leading to a quicker decrease in pH and a stable increase in total acidity. Notably, nitrite accumulation was markedly lower in the FPP group, underscoring FPP’s role in enhancing product safety. FPP also improved antioxidant retention, which is essential for antioxidant capacity and color stability. The use of FPP also enhanced textural integrity, maintaining hardness, chewiness, and fracturability, essential for consumer appeal and extended shelf life. Color stability was another major benefit, as FPP limited oxidative browning and Maillard reaction. Sensory evaluations corroborated these findings, showing consistently superior scores in color, flavor, texture, and taste for FPP pickles. Collectively, FPP is a valuable additive for enhancing the quality characteristics of fermented cowpea pickles, with potential applications in other fermented vegetables. Its ability to increase fermentation efficiency, reduce nitrite levels, retain antioxidants, and preserve texture and color quality aligns with industry trends favoring clean-label, shelf-stable, and health-promoting food products. 

## Figures and Tables

**Table 1:** Changes in the contents of ascorbic acid, total flavonoids, and total phenols during fermentation and storage of cowpea pickles with different treatments.

Treatment	Group	Ascorbic Acid (mg/g)	Total Phenolics (µg/g)	Total Flavonoids (µg/g)
Fermentation 1 d	Control	0.21 ± 0.01 ^Ba^	196.12 ± 2.49 ^Aa^	79.79 ± 1.28 ^Aa^
	Fermentation promoting peptide	0.24 ± 0.01 ^Aa^	210.80 ± 3.24 ^Ba^	83.67 ± 2.66 ^Aa^
Fermentation 31 d	Control	0.14 ± 0.02 ^Bb^	150.43 ± 6.04 ^Ab^	63.42 ± 1.56 ^Bb^
	Fermentation promoting peptide	0.22 ± 0.01 ^Ab^	179.03 ± 1.52 ^Bb^	74.43 ± 1.84 ^Ab^
Accelerated storage 30 d	Control	Not detected.	83.02 ± 6.28 ^Bc^	41.49 ± 2.37 ^Ac^
	Fermentation promoting peptide	Not detected.	97.52 ± 4.78 ^Ac^	43.09 ± 3.52 ^Ac^

Note: Uppercase letters represent significant differences between different groups under the same treatment, while lowercase letters represent significant differences within the same group under different treatments.

**Table 2:** Changes of amino acid nitrogen, total sugar, and 5-HMF contents during fermentation and storage of cowpea pickles.

Treatment	Group	Amino Acid Nitrogen (mg/100 g)	Total Sugar (mg/g)	5-HMF (μg/g)
Fermentation 1 d	Control	112.88 ± 2.80 ^Bb^	121.66 ± 1.80 ^Aa^	Not detected.
	Fermentation promoting peptide	133.56 ± 1.97 ^Ab^	109.59 ± 4.87 ^Ba^	Not detected.
Fermentation 31 d	Control	224.28 ± 7.77 ^Aa^	83.95 ± 1.24 ^Ab^	0.09 ± 0.03 ^Ab^
	Fermentation promoting peptide	182.07 ± 7.13 ^Ba^	64.17 ± 1.26 ^Bb^	0.14 ± 0.02 ^Ab^
Accelerated storage 30 d	Control	11.04 ± 1.54 ^Ac^	8.18 ± 1.09 ^Ac^	24.60 ± 1.08 ^Aa^
	Fermentation promoting peptide	10.73 ± 1.29 ^Ac^	9.03 ± 1.51 ^Ac^	19.76 ± 1.17 ^Ba^

Note: Uppercase letters represent significant differences between different groups under the same treatment, while lowercase letters represent significant differences within the same group under different treatments.

## Data Availability

The data presented in this study are available on request from the corresponding author. The data are not publicly available due to privacy restrictions.

## Supplementary Materials

The following supporting information can be downloaded at: https://www.mdpi.com/article/10.3390/foods13243989/s1, Table S1: Sensory evaluation standard of cowpea pickles.
