# Supplementary material for: Study on the Improvement of Quality Characteristics of Pickles During Fermentation and Storage
Source: Foods. 2024 Dec 10;13(24):3989. doi: 10.3390/foods13243989 (PMC11675974; doi:10.3390/foods13243989)
Supplement: Supplementary file 1 [file foods-13-03989-s001.zip › foods-3315115-supplementary.pdf]

Supplementary Data

List of Supplementary Tables

Table S1. Sensory evaluation standard of cowpea pickles.

Indicators	Evaluation criterion	Score
**Color**	It has a good color, and the color is bright and uniform, and the soaking solution is clear.	80-100
	The color is partially consistent, and the soaking solution is clear.	60-80
	The color is slightly different, and the soaking solution is relatively clear.	40-60
	The color difference is obvious, and the soaking solution is turbid.	20-40
	Abnormal color, impurities or pollutants, soaking liquid turbidity.	0-20
**Flavor**	It has a unique fermentation aroma, and the aroma is rich and coordinated, and the sour taste is suitable.	80-100
	It has a unique fermented fragrance, but the fragrance is not strong, the sour taste is heavy, and it is acceptable.	60-80
	It has a unique fermentation aroma, but the aroma is light, the sour taste is too heavy, and it is difficult to accept.	40-60
	Slightly unique fermentation aroma, but unpleasant odor.	20-40
	No unique fermentation aroma, but a strong unpleasant odor.	0-20
**Texture**	Has excellent fracturability, hardness and chewiness.	80-100
	Has second-rate fracturability, hardness and chewiness.	60-80
	Loses partial fracturability, hardness and chewiness.	40-60
	Soft texture, rougher, less fracturability, hardness and chewiness.	20-40
	Texture is sticky, soft, rough and textureless.	0-20
**Taste**	Taste is best with a strong characteristic taste of cowpea pickles, sour and salty at its best, without any unpleasant tastes.	80-100
	Taste is good, with a slightly weaker taste characteristic of cowpea pickles, suitably sour and salty, with no unpleasant tastes.	60-80
	Taste is ordinary, no characteristic taste of cowpea pickles, sour and salty is not too suitable, slightly unpleasant tastes.	40-60
	Taste acceptance is low, sour and salty are inappropriate, and there are more undesired tastes.	20-40
	Unacceptable taste, too acidic or salty, too many undesired tastes.	0-20
